# Learning Curve of Shape-Sensing Robotic-Assisted Bronchoscopy (ssRAB) for Peripheral Pulmonary Lesions in a Thoracic Surgery Center Using the ION System

**DOI:** 10.3390/jcm15124470

**Published:** 2026-06-09

**Authors:** Donatas Zalepugas, Jan Arensmeyer, Philipp Feodorovici, Mark Coburn, Dirk Skowasch, Tatjana Dell, Julian Luetkens, Joachim Schmidt, Hruy Menghesha

**Affiliations:** 1 Department of Thoracic Surgery, University Hospital Bonn, University of Bonn, Venusberg-Campus 1, 53127 Bonn, Germany; 2 Department of Thoracic Surgery, Helios Clinic Bonn/Rhein-Sieg, 53123 Bonn, Germany; 3 Bonn Surgical Technology Center (BOSTER), University Hospital Bonn, Joseph-Schumpeter-Allee 1, 53227 Bonn, Germany; 4 Department of Anesthesiology and Intensive Care Medicine, University Hospital Bonn, University of Bonn, 53127 Bonn, Germany; 5 Department of Internal Medicine II—Pneumology/Cardiology, University Hospital Bonn, University of Bonn, 53127 Bonn, Germany; 6 Department of Diagnostic and Interventional Radiology, University Hospital Bonn, University of Bonn, 53127 Bonn, Germany

**Keywords:** learning curve, ssRAB, lung nodules, lung cancer screening

## Abstract

**Background**: Robotic-assisted bronchoscopy enables precise navigation to peripheral pulmonary lesions and expands minimally invasive diagnostic options in thoracic surgery. At our institution, the ION™ Endoluminal System (Intuitive Surgical, Sunnyvale, CA, USA) was introduced to improve diagnostic accuracy in challenging peripheral targets. It is widely recognized that a defined number of procedures is required to achieve procedural proficiency and optimal clinical outcomes when adopting a novel platform. Therefore, this retrospective single-center study aimed to evaluate the learning curve associated with the implementation of this technology in a thoracic surgery center. **Methods**: In this retrospective study, all consecutive patients who underwent robotic-assisted bronchoscopies performed using the ION™ Endoluminal System (Intuitive Surgical, Sunnyvale, CA, USA) for the diagnosis of peripheral pulmonary lesions between August 2024 and March 2026 were analyzed. A total of 128 lesions in 89 patients were initially identified. Cases involving marker placement without diagnostic biopsy, as well as procedures not performed by the primary operator, were excluded. After applying exclusion criteria, 109 procedures in 76 patients were included. The mean patient age was 65.4 ± 9.1 years, and 44 patients were female (57.9%). To assess the learning curve, procedures were chronologically divided into three groups: early (cases 1–36), intermediate (37–73), and late (74–109). Outcome measures included procedure time, number of biopsies per lesion, tumor size, and diagnostic yield. Group comparisons were performed using non-parametric and chi-square tests. Procedural learning was assessed by cumulative sum (CUSUM) analysis of procedure time. **Results**: The overall diagnostic yield was 85.3% (93/109). The diagnostic yield increased over time from 73.0% in the early phase to 83.3% in the intermediate phase and 94.6% in the late phase. The overall comparison was statistically insignificant (χ^2^ *p* = 0.117); however, there was a significant linear trend across phases, indicating progressive improvement with exposure to the application of this technology. Procedure time decreased significantly from a median of 49.0 min in the early phase to 31.0 min in the intermediate phase and 30.0 min in the late phase (*p* < 0.001). At the same time, the number of biopsies per lesion increased significantly (*p* < 0.001). Tumor size did not differ significantly between groups (*p* = 0.170). **Conclusions**: Robotic-assisted bronchoscopy demonstrates a clear learning curve, characterized by increasing diagnostic yield and significantly reduced procedure time during the implementation phase. The technique can be effectively integrated into the thoracic surgical diagnostic workflow and represents a valuable addition to minimally invasive diagnostics for peripheral pulmonary lesions.

## 1. Introduction

The increasing detection of small peripheral pulmonary lesions, driven by the widespread use of high-resolution computed tomography and lung cancer screening programs, has created a growing demand for accurate and minimally invasive diagnostic techniques. While conventional bronchoscopy remains limited in its ability to reach peripheral targets, transthoracic needle biopsy is associated with a higher risk of complications, including pneumothorax and bleeding in particular. Therefore, advanced bronchoscopic navigation techniques have emerged to improve diagnostic yield while maintaining a favorable safety profile [1,2,3].

New technologies, including electromagnetic navigation bronchoscopy (ENB) and radial endobronchial ultrasound (rEBUS), have been developed in the last several years to improve the ability to access peripheral lesions. Although these methods have been found useful for increasing the diagnostic yield, they still exhibit considerable variability and depend heavily on both the characteristics of the lesion and the skill of the operator performing the procedure [4,5,6]. Consequently, further technological advances have been pursued to overcome these limitations.

The ION™ Endoluminal System (Intuitive Surgical, Sunnyvale, CA, USA) employs shape-sensing technology that provides a real-time tracking capability to locate and navigate toward peripheral targets, ultimately providing better diagnostic accuracy for difficult-to-sample lesions [7,8,9]. Early studies using the ION have shown promising results in terms of diagnostic yield and safety; however, there is still a limited number of studies published that demonstrate its use in thoracic surgical settings [10,11,12].

Additionally, as with all new technologies, the successful use of the ION System is influenced by the learning curve associated with the use of robotic-assisted bronchoscopy, leading to differences in procedural efficiency, diagnostic yield, and overall workflow. By understanding the learning curve for the procedures, we may optimize training, allocate resources properly, and improve clinical outcomes. While initial reports have described feasibility and performance outcomes, there remains limited evidence on the procedural learning process, especially outside of high-volume interventional pulmonology centers [13,14,15].

Kemp et al. demonstrated the existence of a learning curve for endobronchial ultrasound using cumulative sum (CUSUM) analysis, showing that procedural competency improves with experience. More recently, Mullin et al. reported operator learning effects during robotic-assisted bronchoscopic biopsy of pulmonary nodules, suggesting improvements in diagnostic performance over time. However, most available data originate from interventional pulmonology programs, and evidence regarding the implementation of robotic-assisted bronchoscopy within thoracic surgery departments remains scarce. Therefore, the transferability of existing learning curve data to thoracic surgical practice remains uncertain.

The aim of the present study was to evaluate the learning curve associated with the implementation of robotic-assisted bronchoscopy using the ION system for the diagnosis of peripheral pulmonary lesions in a thoracic surgery center. We hypothesize that a measurable learning curve exists during the adoption of shape-sensing robotic-assisted bronchoscopy, characterized by progressive improvements in diagnostic yield and procedural efficiency with increasing operator experience.

## 2. Materials and Methods

### 2.1. Study Design and Patient Selection

This retrospective cohort study included all consecutive patients undergoing robotic-assisted bronchoscopy using the ION™ Endoluminal System (Intuitive Surgical, Sunnyvale, CA, USA) for the diagnosis of peripheral pulmonary lesions at a tertiary thoracic surgical center between August 2024 and March 2026. All adult patients referred for bronchoscopic evaluation of pulmonary nodules were considered eligible.

The study was approved by the institutional ethics committee (2026-205-BO) and conducted in accordance with the ethical principles of the Declaration of Helsinki. All data were obtained from the institution’s electronic medical records and were fully anonymized prior to analysis. Due to the retrospective design and anonymization of data, the requirement for individual informed consent was waived in accordance with applicable regulations.

This retrospective cohort study was registered in the German Clinical Trials Register (DRKS; registration number: DRKS00039920).

### 2.2. Exclusion Criteria

**Figure 1:**
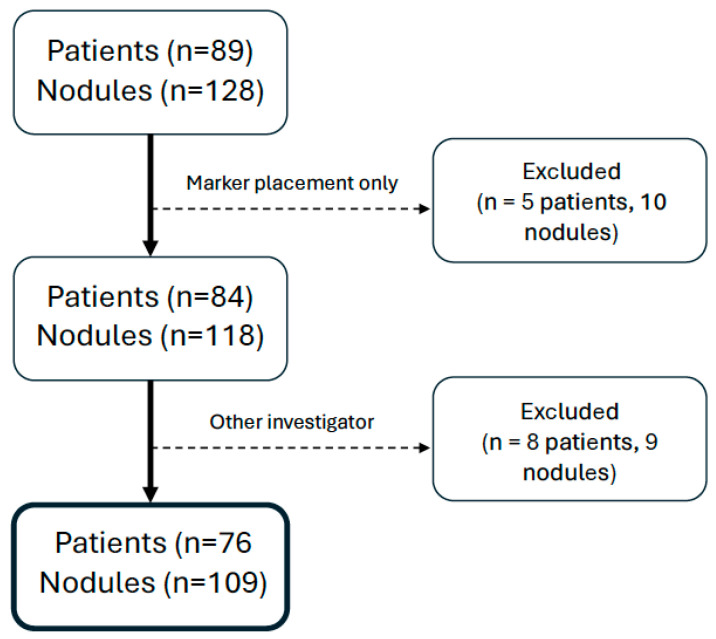
Study flow diagram of patient and nodule selection: flow diagram illustrating the selection process of patients and pulmonary nodules included in the study. Exclusion criteria and the final cohort are shown.

Patients undergoing procedures for marker placement only (*n* = 5 patients, 10 nodules) without diagnostic biopsy were excluded. In addition, procedures not performed by the primary operator (*n* = 8 patients, 9 nodules) were excluded to ensure consistency in the assessment of the learning curve (Figure 1).

### 2.3. Preoperative Preparation

All patients with pulmonary nodules identified on chest computed tomography (CT) underwent structured evaluation based on lesion characteristics and estimated malignancy risk. Each case was discussed in a multidisciplinary tumor board, and indications for tissue diagnosis were determined by consensus. Preoperative assessment included a detailed medical history, physical examination, and functional evaluation according to current clinical guidelines. This included pulmonary function testing with body plethysmography and measurement of diffusing capacity for carbon monoxide (DLCO), a 12-lead electrocardiogram, and routine laboratory testing. Additional diagnostic investigations, such as transthoracic echocardiography or cardiopulmonary exercise testing, were performed when clinically indicated.

### 2.4. Procedural Workflow

All procedures were performed under general anesthesia with endotracheal intubation and continuous intraoperative monitoring by an attending anesthesiologist. All robotic-assisted bronchoscopies included in this analysis were conducted by a single experienced thoracic surgeon using the ION™ system.

Preprocedural planning was performed using the PlanPoint™ software Version OS1v5.1.1.2f65f5f04 (Intuitive Surgical, Sunnyvale, CA, USA), which generates CT-based virtual airway maps to facilitate navigation to peripheral lesions. In all cases, lesion localization and confirmation of tool-in-lesion were achieved using cone-beam computed tomography (CBCT), ensuring a standardized imaging-guided approach.

Biopsy instruments were selected at the operator’s discretion based on lesion characteristics and included needle aspiration, forceps biopsy, cryobiopsy, or a combination of these techniques. Intraoperative frozen section analysis was performed when clinically indicated. In cases of confirmed or highly suspected malignancy, subsequent surgical resection was performed during the same anesthetic session, where appropriate.

### 2.5. Histopathological Evaluation

Tissue samples obtained during robotic-assisted bronchoscopy were either subjected to intraoperative frozen section analysis or processed for definitive histopathological evaluation, depending on the clinical context. Specimens were processed using standard formalin fixation and paraffin embedding (FFPE) protocols. Final diagnoses were established according to the current World Health Organization (WHO) classification of thoracic tumors.

### 2.6. Diagnostic Yield

The definition of diagnostic yield was adopted according to the consensus statement of the American Thoracic Society and the American College of Chest Physicians. In this context, diagnostic yield is defined as “the proportion of all individuals undergoing the diagnostic procedure under evaluation in whom a specific malignant or benign diagnosis is established.” Accordingly, procedures yielding a specific malignant or benign histopathological diagnosis were classified as diagnostic, whereas procedures resulting in normal lung tissue, insufficient material, blood, or non-specific findings without a definitive pathological diagnosis were classified as non-diagnostic [16].

### 2.7. Outcome Measures

The primary outcome of this study was diagnostic yield. To evaluate the learning curve, procedures were chronologically divided into three groups: early phase (cases 1–36), intermediate phase (cases 37–73), and late phase (cases 74–109).

Secondary outcome measures included procedure time, defined as the duration from bronchoscope insertion to procedure completion, the number of biopsy samples obtained per lesion, and tumor size as measured on preprocedural CT imaging.

### 2.8. Statistical Analysis

Continuous variables are presented as median and range. Comparisons between the three procedural phases were performed using the Kruskal–Wallis test. When statistically significant differences were identified, pairwise comparisons were conducted using the Mann–Whitney U test with Bonferroni correction for multiple testing. Categorical variables were compared using the chi-squared test. A two-sided *p*-value < 0.05 was considered statistically significant. Statistical analyses were performed using IBM SPSS Statistics version 29 (IBM Corp., Armonk, NY, USA).

To further evaluate procedural learning as a continuous process, a cumulative sum (CUSUM) analysis of procedure time was performed. For each procedure, the deviation from the overall mean procedure time was calculated and cumulatively summed in chronological order. The resulting CUSUM curve was used to visualize changes in procedural efficiency over time and to identify phases of learning and stabilization.

## 3. Results

### 3.1. Patient and Lesion Characteristics

A total of 109 nodules in 76 patients were included in the final analysis. The mean age was 65.4 ± 9.1 years, and 44 patients (57.9%, 44/76) were female. Regarding smoking status, 25 patients (32.9%, 25/76) were current smokers, 30 (39.5%, 30/76) were former smokers, and 21 (27.6%, 21/76) had never smoked. The mean cumulative smoking exposure was 31.8 ± 16.3 pack-years. The mean body mass index was 27.3 ± 5.8 kg/m^2^. Most lesions were located in the peripheral third of the lung (82.6%, 90/109), followed by the middle third (13.8%, 15/109) and central third (3.7%, 4/109). Lesions were most frequently found in the right upper lobe (35.7%, 39/109) and left upper lobe (25.7%, 28/109), followed by the left lower lobe (24.8%, 27/109), right lower lobe (8.3%, 9/109), and middle lobe (5.5%, 6/109). The mean lesion size was 11.6 ± 7.6 mm. The majority of lesions were solid (79.8%, 87/109), while 8.3% (9/109) were subsolid and 11.9% (13/109) presented as ground-glass opacities (Table 1).

### 3.2. Procedural Characteristics and Overall Outcomes

Cone-beam computed tomography (CBCT) was used in 102 procedures (93.6%, 102/109), while C-arm fluoroscopy alone was used in 7 cases (6.4%, 7/109). The most frequently used biopsy modality was cryobiopsy (59.6%, 65/109), followed by forceps biopsy (30.3%, 33/109), combined cryobiopsy and forceps (8.3%, 9/109), and needle aspiration (1.8%, 2/109). The mean number of biopsies per lesion was 6.6 ± 2.7, and the mean procedure time was 39.9 ± 18.6 min. The overall diagnostic yield was 85.3% (93/109). Sixteen procedures were classified as non-diagnostic. Of these, 12 patients subsequently underwent surgical resection and obtained a definitive histopathological diagnosis, whereas four patients were managed non-operatively with multidisciplinary clinical assessment and radiological follow-up. One minor bleeding event (0.9%, 1/109) occurred and was managed conservatively. No pneumothorax occurred during the study period. No serious adverse events were observed (Table 1).

### 3.3. Learning Curve Analysis

#### 3.3.1. Diagnostic Yield

**Figure 2:**
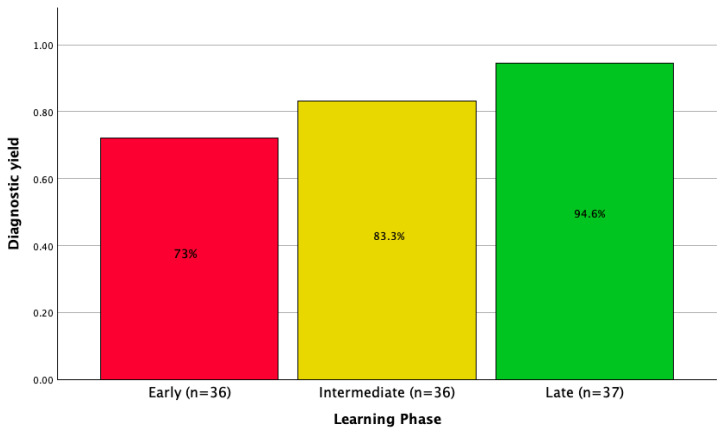
Bar chart of diagnostic yield by group.

Diagnostic yield increased across the three phases, from 73.0% (approximately 26/36) in the early phase to 83.3% (30/36) in the intermediate phase and 94.6% (35/37) in the late phase. Although the overall comparison did not reach statistical significance (*p* = 0.117), a continuous upward trend was observed (Table 2, Figure 2).

#### 3.3.2. Procedure Time

**Figure 3:**
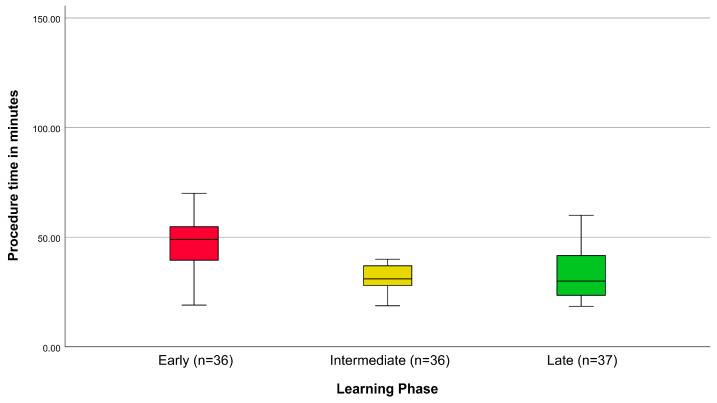
Box plot of procedure time by group.

Procedure time differed significantly across the three phases (Kruskal–Wallis test, *p* < 0.001). Median procedure time was 49.0 min [19.0;88.0] in the early phase, compared to 31.0 min [18.8;62.0] in the intermediate phase and 30.0 min [18.5;135.0] in the late phase (*p* = 0.012) (Table 2, Figure 3). Pairwise comparisons with Bonferroni correction demonstrated significantly longer procedure times during the early phase compared with both the intermediate (adjusted *p* < 0.001) and late phases (adjusted *p* = 0.001), whereas no significant difference was observed between the intermediate and late phases (adjusted *p* = 1.000).

#### 3.3.3. Number of Biopsies

**Figure 4:**
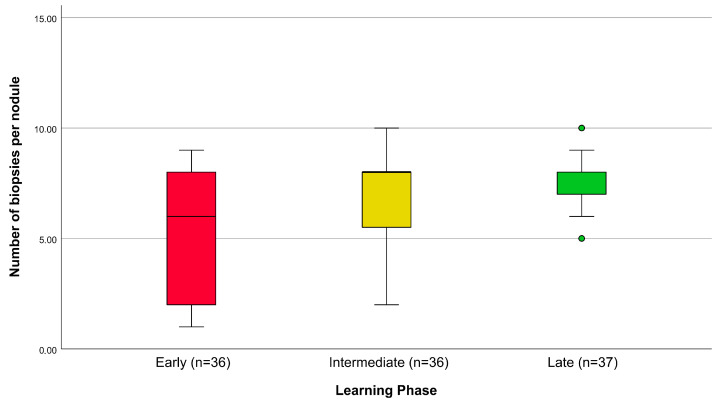
Box plot of number of biopsies by group.

The number of biopsy samples obtained per lesion differed significantly across the three phases (Kruska–Wallis test, *p* < 0.001). Median values rose from 6.0 [1;9] in the early phase to 8.0 [2;10] in the intermediate phase and remained stable at 8.0 [2;13] in the late phase (Table 2, Figure 4). Pairwise comparisons with Bonferroni correction demonstrated significantly fewer biopsy samples during the early phase compared with both the intermediate (adjusted *p* = 0.021) and late phases (adjusted *p* < 0.001). No significant difference was observed between the intermediate and late phases (adjusted *p* = 0.290).

#### 3.3.4. Tumor Size

Tumor size did not differ significantly between the three groups (*p* = 0.170), indicating comparable lesion characteristics across the learning phases (Table 2).

#### 3.3.5. CUSUM Analysis of Procedure Time

**Figure 5:**
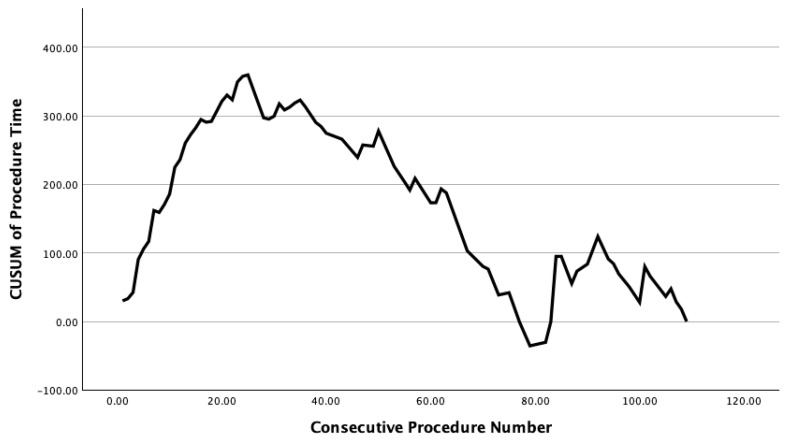
Cumulative sum (CUSUM) analysis of procedure time.

CUSUM analysis demonstrated a characteristic learning curve pattern (Figure 5). During the initial implementation phase, the CUSUM curve increased and reached a maximum after approximately 25 procedures, indicating procedure times above the overall mean. Thereafter, the curve showed a sustained downward trend, reflecting progressive improvement in procedural efficiency. In the later phase of the series, the curve stabilized around the reference level, suggesting increasing procedural consistency and attainment of procedural proficiency.

## 4. Discussion

### 4.1. Principal Findings

In this study, we evaluated the learning curve associated with the implementation of robotic-assisted bronchoscopy using the ION system in a thoracic surgery setting. The main findings can be summarized as follows: first, diagnostic yield increased progressively across the three learning phases, reaching 94.6% in the late phase; second, procedure time decreased significantly over time; and third, the number of biopsies per lesion increased significantly, while lesion characteristics remained comparable across groups. Together, these findings demonstrate a clear learning curve characterized by improved efficiency and diagnostic performance.

### 4.2. Learning Curve and Procedural Efficiency

The observed reduction in procedure time represents one of the most robust indicators of procedural learning. The median procedure time decreased from 49 min in the early phase to approximately 30 min in the intermediate and late phases, reflecting rapid familiarization with the system and increasing procedural confidence. This improvement is consistent with prior studies on advanced bronchoscopic techniques, demonstrating that procedural efficiency improves significantly after an initial learning phase [9,17].

Importantly, the most pronounced reduction occurred between the early and intermediate phases, suggesting that a substantial portion of the learning curve is completed within the first 30–40 cases. This observation has practical implications for training programs and resource planning. This interpretation is further supported by the non-parametric analyses, which demonstrated significant differences between the early phase and both subsequent phases, whereas no significant differences were observed between the intermediate and late phases.

The additional CUSUM analysis further supports this observation by demonstrating the continuous nature of procedural learning. The CUSUM curve increased during the initial implementation phase and reached its maximum after approximately 25 procedures, indicating procedure times above the overall mean. Thereafter, the curve showed a sustained decline and eventual stabilization, reflecting progressive improvement in procedural efficiency and increasing operator familiarity with the platform. These findings suggest that the greatest gains in efficiency occur during the early phase of adoption, followed by a period of procedural consolidation.

Our findings are consistent with those reported by Mullin et al., who demonstrated improved procedural efficiency with increasing experience and achievement of proficiency after 18–36 procedures. Similarly, our CUSUM analysis showed the greatest improvement during the first approximately 25 procedures, with subsequent stabilization of performance. Procedure time also decreased substantially from 49 min in the early phase to 30 min in the late phase, supporting the presence of a clinically relevant learning curve.

Interestingly, procedural efficiency improved despite an increase in the number of biopsy samples obtained per lesion. While the median number of biopsies increased from six to eight, procedure time decreased substantially from 49 to 30 min. As obtaining additional samples contributes only minimally to overall procedure duration once the target lesion has been reached, this finding suggests that improved navigation efficiency and increasing operator familiarity with the robotic platform were the primary drivers of the observed reduction in procedure time.

### 4.3. Diagnostic Yield and Clinical Performance

Although the increase in diagnostic yield did not reach statistical significance in the overall comparison, a clear upward trend was observed, supported by a significant linear association. The lack of statistical significance is most likely attributable to limited statistical power rather than the absence of a true effect.

The diagnostic yield observed in this study is consistent with recent literature. A meta-analysis by Zhang et al. reported a pooled diagnostic yield of approximately 80% for robotic-assisted bronchoscopy [9]. More recent studies have demonstrated yields ranging from 75% to 87%, depending on lesion characteristics and procedural setup [17,18]. Notably, the diagnostic yield observed in the late phase of the present study (94.6%) exceeds these values and is likely related to increasing operator experience and the standardized use of cone-beam CT.

The integration of CBCT has been shown to further improve diagnostic performance by enabling real-time confirmation of tool-in-lesion and reducing CT-to-body divergence, a known limitation of conventional navigation techniques [19,20].

### 4.4. Biopsy Strategy and Sampling Behavior

An important finding of this study is the significant increase in the number of biopsies per lesion over time. This likely reflects an evolution in operator strategy, with more systematic and comprehensive sampling performed as experience increased.

Previous studies have demonstrated that the number and quality of biopsy samples are key determinants of diagnostic success in bronchoscopic procedures [21,22]. The combination of improved navigation accuracy and optimized sampling strategy may therefore contribute synergistically to the observed increase in diagnostic yield.

### 4.5. Comparison with Existing Technologies

Robotic-assisted bronchoscopy has been developed to overcome the limitations of conventional bronchoscopy and earlier navigation systems. Diagnostic yield for conventional and guided bronchoscopy remains highly variable, typically ranging between 40% and 70%, particularly for small peripheral lesions [23,24]. Electromagnetic navigation bronchoscopy (ENB), while representing a significant advancement, has reported pooled yields of approximately 63–65% [4,25].

In contrast, robotic-assisted bronchoscopy offers enhanced stability, improved distal reach, and precise instrument control, translating into improved and more consistent diagnostic performance [26,27]. The results of the present study support these advantages and demonstrate that a high diagnostic yield can be achieved in a real-world clinical setting.

### 4.6. Implementation in a Thoracic Surgery Setting

A distinctive aspect of this study is the implementation of robotic-assisted bronchoscopy within a thoracic surgery department. Most previously published studies have been conducted in interventional pulmonology settings. Our findings demonstrate that this technology can be effectively integrated into a surgical workflow.

This integration may facilitate streamlined decision-making, allow for single-stage diagnostic and therapeutic procedures, and improve coordination within multidisciplinary care pathways. These factors may contribute to improved efficiency and patient outcomes, particularly in patients with suspected malignancy.

### 4.7. Safety Profile

The procedure demonstrated an excellent safety profile, with no serious adverse events and only one minor bleeding event (0.9%). These findings are consistent with previously published data reporting low complication rates for robotic-assisted bronchoscopy [28,29].

The minimally invasive nature of the technique, combined with improved navigation control and visualization, likely contributes to its favorable safety profile compared to transthoracic biopsy approaches.

### 4.8. Limitations

Several limitations should be acknowledged. First, the retrospective single-center design may limit the generalizability of our findings. In addition, the procedural volume at our institution was approximately five to six robotic bronchoscopy procedures per month; therefore, the learning curve observed in this study may not be directly transferable to lower-volume centers performing substantially fewer procedures.

Second, the sample size was relatively small, which may have limited statistical power, particularly for detecting differences in diagnostic yield. Third, all procedures were performed by a single operator. While this allowed for a consistent assessment of the learning curve, exclusion of the small number of procedures performed by other operators may have introduced a potential selection bias and may limit the applicability of our findings to operators with different levels of experience or training backgrounds.

Furthermore, potential confounding factors, including the bronchus sign, subtle differences in lesion morphology, and anatomical accessibility, were not fully accounted for. Finally, the division of procedures into three phases represents a simplified approach to learning curve assessment and may not fully capture the continuous and dynamic nature of procedural skill acquisition.

### 4.9. Conclusions

Robotic-assisted bronchoscopy using the ION system demonstrates a clear and clinically relevant learning curve in a thoracic surgery setting. Procedural efficiency improves rapidly, while diagnostic performance increases progressively with experience. The technique can be safely and effectively integrated into routine clinical practice and represents a valuable addition to minimally invasive diagnostics for peripheral pulmonary lesions.

## Figures and Tables

**Table 1:** Baseline characteristics.

	Total *n* = 76 Patients *n* = 109 Nodules
Patient characteristics	
Age (years)	65.39 ± 9.14
Female (%)	44 (57.89%)
Smoking status (%)	
current	25 (32.9%)
former	30 (39.5%)
never	21 (27.6%)
Pack-years	31.8 ± 16.3
BMI (kg/m^2^)	27.34 ± 5.79
Lesion characteristics	
Topographical data	
Peripheral third	90 (82.6%)
Middle third	15 (13.8%)
Central third	4 (3.7%)
Lesion Lobe	
RUL	39 (35.7%)
ML	6 (5.5%)
RLL	9 (8.3%)
LUL	28 (25.7%)
LLL	27 (24.8%)
Lesion size (mm)	11.62 ± 7.64
Lesion Density	
Solid	87 (79.8%)
Sub solid	9 (8.3%)
GGO	13 (11.9%)
Procedural characteristics	
Imaging tool	
CBCT	102 (93.6%)
C-arm	7 (6.4%)
Biopsy tool (nodules)	
Needle	2 (1.8%)
Forceps	33 (30.3%)
Cryoprobe	65 (59.6%)
Cryoprobe + Forceps	9 (8.3%)
Number of biopsies per nodule	6.59 ± 2.68
Procedure time (min.)	39.9 ± 18.6
Diagnostic yield (%)	85.3
(Serious) Adverse Event	0%
Minor bleeding	1 (0.9%)

**Table 2:** Evolution of procedural performance and diagnostic outcomes across learning phases.

	Early (*n* = 36)	Intermediate (*n* = 36)	Late (*n* = 37)	*p*-Value
Diagnostic Yield (%)	73.0	83.3	94.6	0.117
Procedure time (min.)	49.0 [19;88]	31.0 [18.8;62.0]	30.0 [18.5;135.0]	<0.001
Number of biopsies	6.0 [1;9]	8.0 [2;10]	8.0 [2;13]	<0.001
Tumor size (mm)	10.5 [3;36]	10.0 [3;24]	8.5 [3;30]	0.170

## Data Availability

The datasets generated during and/or analyzed during the current study are not publicly available due to security and ongoing research. The data underlying this article will be shared upon reasonable request from the corresponding author.
